# Assessment of the Immune Potential of Calcium Phosphate-Outer Membrane Protein-Nanoparticles (CaP-Omp-Nps) Adjuvanted Nano-Vaccine Against *Salmonella* Typhimurium in Poultry Birds

**DOI:** 10.3390/pharmaceutics18060681

**Published:** 2026-05-29

**Authors:** Anjani Saxena, Yashpal Singh, Mumtesh Kumar Saxena, Sachin Kumar, Meena Mrigesh, Aman Kamboj, Manish Kumar Verma, Manjul Kandpal, Satya PalSingh

**Affiliations:** 1 Department of Veterinary Pharmacology & Toxicology, College of Veterinary & Animal Sciences, G.B. Pant University of Agriculture & Technology, Pantnagar 263145, Uttarakhand, India; 2 Department of Animal Genetics & Breeding, College of Veterinary & Animal Sciences, G.B. Pant University of Agriculture & Technology, Pantnagar 263145, Uttarakhand, India; 3 Centre for Biomedical Engineering, Indian Institute of Technology, Delhi 110016, India; 4 Department of Veterinary Anatomy, College of Veterinary & Animal Sciences, G.B. Pant University of Agriculture & Technology, Pantnagar 263145, Uttarakhand, India; 5 Department of Veterinary Clinical Complex, College of Veterinary & Animal Sciences, G.B. Pant University of Agriculture & Technology, Pantnagar 263145, Uttarakhand, India; 6 Department of Veterinary Surgery and Radiology, College of Veterinary & Animal Sciences, G.B. Pant University of Agriculture & Technology, Pantnagar 263145, Uttarakhand, India

**Keywords:** calcium phosphate nanoparticles, outer membrane proteins, poultry, vaccine, immunity, non-typhoidal salmonellosis

## Abstract

**Background***: Salmonella* Typhimurium is a major pathogen causing non-typhoidal salmonellosis in humans. Poultry is a major reservoir of S. Typhimurium. Currently available vaccines against S. Typhimurium are not very effective. Therefore, the search for novel adjuvants to improve vaccine efficacy is a priority for developing effective and efficient vaccines. **Method**: In this study, next-generation adjuvants, such as calcium phosphate nanoparticles, are being evaluated. Our objective was to assess the potential of calcium phosphate nanoparticles, using outer membrane proteins of *Salmonella* Typhimurium as antigens, for immune-potential testing in poultry, with Montanide as a control. The toxicity of the prepared vaccine formulation was evaluated in rats. **Results:** CaP-Omp-Nps in the 30–45 nm size range showed a protein entrapment efficiency of 42.5% and a loading capacity of 50.3%. Both vaccinated groups, calcium phosphate outer membrane protein nanoparticles (CaP-Omp-Nps) and Montanide, induced an efficient humoral immune response, with mean titers of 3.48 + 0.0245 and 4.9 + 0.0142 on the 15th day, 3.5 + 0.0118 and 4.79 + 0.009 on the 30th day, and 4.48 + 0.427 and 5.31 + 0.154 on the 45th day post vaccination, respectively, indicating an improvement (CaP-Omp-Nps group) or stability (Montanide group) over the study period. Further, the CaP-Omp-Nps group revealed a better cell-mediated immune response than the Montanide-Omp group. The toxicity study in rats showed no significant differences in serum biomarkers and blood chemistry parameters, indicating that the nano-vaccine formulation is non-toxic and safe. Outer membrane proteins of *Salmonella* Typhimurium, when used with a few conventional adjuvants, could not produce a balanced Th1 and Th2 immune response against *Salmonella* Typhimurium. **Conclusions:** In this study, we developed a novel nano-vaccine formulation composed of outer membrane proteins of *Salmonella* Typhimurium and calcium phosphate nanoparticles. The vaccine formulation was found to be safe and could elicit the desired Th1 and Th2 immune responses, as evidenced by humoral, cell-mediated, and protective immunity produced by the nano vaccine in poultry. Therefore, the present findings suggest that the CaP-Omp-Nps vaccine may be an efficient, safe, and cost-effective vaccine against *Salmonella* Typhimurium.

## 1. Background

Non-typhoidal salmonellosis is a significant foodborne and waterborne disease affecting both humans and animals. *Salmonella* Typhimurium, a major serovar of Salmonella, causes non-typhoid salmonellosis. In humans, the disease manifests as gastroenteritis, but severe complications may occur in some cases [1]. Annually, 535,000 cases and over 75,000 deaths are attributed to non-typhoidal salmonellosis, with the highest mortality rates reported in children under 5 in sub-Saharan Africa and Asia [2]. While antibiotics are commonly used to treat gastroenteritis, rising antibiotic resistance in *Salmonella* complicates treatment [3]. Consequently, vaccination is considered the most promising control and eradication method at present. Existing vaccines include killed and live mutant types, but each has limitations: live vaccines pose safety risks and may revert to virulence, whereas the ability of killed vaccines to induce cell-mediated immunity remains questionable [4]. There is an urgent need for improved vaccines containing immunogenic antigens and effective adjuvants. Outer membrane proteins (Omps) of *Salmonella* have demonstrated immune potential in previous studies and are conserved among serovars [5,6,7,8]. In other pathogenic bacteria, Omps have also elicited protective immune responses [9]. Adjuvants, crucial to vaccine efficacy, have included alum, aluminum hydroxide, and saponin, but sometimes fail to elicit a balanced Th1 and Th2 immune response, essential for adequate protection [10,11,12]. Advances in nanotechnology have led to the development of nano-adjuvants like chitosan, silver, and calcium phosphate nanoparticles, which enhance antigen delivery and can induce tailored immune responses: Th 1, Th 2, or balanced [13,14]. Previously, our research group used CaP-Omp-Nps of *S.* Typhi, which successfully elicited strong immune responses in mice and proved safe in toxicity tests [15]. Building on this; our current research focuses on *S*. Typhimurium Omps conjugated to calcium phosphate nanoparticles, evaluating their immunological effects in poultry, a common source of *S*. Typhimurium infection in humans. In earlier studies, calcium phosphate nanoparticles were used to deliver nucleic acids or anticancer drugs. In our study, we used whole outer membrane proteins of *Salmonella* Typhimurium as the antigen, adjuvanted with calcium phosphate nanoparticles. The nano vaccine formulation was extensively characterized for biophysical properties such as nanoparticle size, shape, stability, purity, antigen entrapment efficiency, and antigen release efficacy. The nano-vaccine formulation was analyzed for purity and functional groups [16]. The vaccine was tested in Wistar rats (a lab animal model recommended by WHO) for toxicity analysis. The immunogenic potential of the vaccine was further tested in poultry (host species) to analyze humoral, cell-mediated, and protective immunity of the nano-vaccine formulation. The novelty of the study is that, to the best of our knowledge, it is the first study with Omps of *Salmonella* Typhimurium adjuvanted with calcium phosphate nanoparticles, which includes the synthesis of a nano-vaccine formulation, biophysical characterization, toxicity analysis, and testing of immune potential in poultry birds (host species). The hypothesis of the study was that outer membrane proteins are immunogenic and conserved among the serovars of *Salmonella.* Use of outer membrane protein as an antigen can overcome the problem of vaccination failure due to genetic variation among the field isolates. Calcium phosphate nanoparticles as an adjuvant can induce balanced Th 1, Th 2 immune responses. Therefore, a combination of outer membrane proteins and calcium phosphate nanoparticles may produce a desirable protection against *Salmonella* Typhimurium in poultry birds. The basic objectives of this study were to evaluate the nano vaccine’s safety and its capacity to induce humoral, cell-mediated, and protective immune responses, with the aim to explore the possibility of the development of an effective nano-vaccine against poultry salmonellosis.

## 2. Methods

### 2.1. Bacterial Strains, Culture Media, and Experimental Animals

The *Salmonella* Typhimurium (MTCC 3321) reference strain was procured from the Indian Institute of Microbial Technology, Chandigarh, and maintained in Luria-Bertani broth (Himedia, Mumbai, India) and in 50% glycerol stock. Lab animals (Wistar rats, 6–8 weeks old) were procured from the Indian Veterinary Research Institute, Izatnagar, India, and reared in accordance with the institutional animal ethics committee guidelines. Specific pathogen-free (SPF) poultry eggs obtained from Venky’s India Ltd., Pune, India, were hatched at the Institutional poultry farm, G.B. Pant University of Agriculture and Technology, Pantnagar. The chicks and birds were maintained in experimental rooms of the Department of Microbiology, College of Veterinary and Animal Sciences, under strict hygienic conditions.

### 2.2. Formulation of Calcium Phosphate Adjuvanted Nanoparticles

Outer membrane proteins (Omps) were isolated from the *S.* Typhimurium reference strain (MTCC 3321) by the ultracentrifugation method [17]. In brief, a single colony of *S*. Typhimurium from a Hektoen Enteric Agar plate was inoculated into 5 mL of Luria–Bertani broth and incubated for 18 h in an incubator shaker at 37 °C. The culture was subcultured into 1 L of Luria–Bertani broth and incubated at 37 °C for 18 h in an incubator shaker. The culture was pelleted by centrifugation at 9000× *g* for 10 min at 4 °C in a refrigerated centrifuge (Sigma 18K, USA). The pellet was washed twice with phosphate buffer saline (PBS, pH 7.4) and re-suspended in 10 mM HEPES buffer (pH 7.4). The suspended pellet was sonicated (20 cycles, 7.0 µ, 60 s (followed by 30 s pause) and centrifuged at 1700× *g* for 20 min using an ultra sonicator (Sanyo, Japan). The supernatant was collected and centrifuged at 100,000× *g* for 60 min at 4 °C in an Ultra centrifuge (Sorvall WX Ultra, USA). The pellet obtained was suspended in 2 mL of 2%. N-lauryl sarcosine sodium salt, in 10 mM HEPES buffer, followed by incubation at 37 °C for two hours, followed by ultracentrifugation at 10,000× *g* for 60 min. Finally, the Omps pellet was resuspended in PBS (pH 7.2), lyophilized using lyophilizer (Christ Alpha 1-2, Germany) and stored at −80 °C. Omps were quantified using Lowry’s method and characterized by SDS-PAGE [18,19]. CaP-Omp-Nps were synthesized by the co-precipitation method [20]. In brief, Omps were resuspended in PBS (pH 7.4), and 1 mg/mL of Omps was used for a single preparation. One ml of Omps preparation (1 mg/mL) was added to a 250 mL flask with a magnetic bar in stirring mode (300 rpm) on a magnetic stirrer. Then, 7.5 mL of calcium chloride (12.5 mM) was added drop-by-drop, followed by the addition of 15 mL of sodium citrate (15.6 mM). Furthermore, 7.5 mL of dibasic sodium phosphate (12.5 mM) was added dropwise, and the suspension was stirred for 1 h and then centrifuged at 800× *g* for 20 min at 4 °C. The supernatant was discarded, and the pellet was washed with 0.1 M phosphate buffer saline (PBS, pH 7.4). Finally, pellets composed of calcium-phosphate-adjuvanted Omps nanoparticles were lyophilized and stored at −20 °C for subsequent use. The protein entrapment efficiency of the vaccine formulation was determined by releasing Omps from the nano-adjuvanted complex [20].

### 2.3. Characterization of Calcium Phosphate Adjuvanted Outer Membrane Protein Nanoparticles (CaP-Omp-Nps)

CaP-Omp-Nps were characterized for their shape, size, and stability. The size of nanoparticles was determined by using transmission electron microscopy (TEM) and dynamic light scattering (DLS). For transmission electron microscopy, a TEM grid was coated with 10 µL of a nanoparticle suspension in sterilized water. After air-drying for 5 min, the grid was stained with 2% uranyl acetate and imaged using a transmission electron microscope (JEOL-1011). In DLS, the lyophilized nanoparticle powder was suspended in sterilized water and then ultrasonicated for 30 min. The size was determined using a Zetasizer Nano ZS90 (Malvern Instruments, Malvern, UK). The purity and functional groups of the nano-vaccine formulation were analyzed using energy-dispersive X-ray spectroscopy (EDX) and Fourier Transform Infrared (FTIR) spectroscopy of calcium phosphate-adjuvanted nanoparticles [16].

### 2.4. Preparation of Nano-Vaccine Formulation

Calcium phosphate-Omps adjuvanted nanoparticles were coated with cellobiose and an outer layer of Omps, following the established protocol [21]. In brief, CaP-Omp-Nps were re-suspended in 129 mM of cellobiose dissolved in 0.1 M phosphate buffer saline (pH 7.2) and incubated at 4 °C overnight. During cellobiose coating, the free hydroxyl groups of cellobiose and the reactive charged groups of calcium phosphate interact, stabilizing the coating and producing a depot effect that promotes the slow release of antigen in the host body. Later, 3 mg of Omps was added to the coated nanoparticles, and the mixture was incubated for 1 h at 4 °C. The suspension was centrifuged at 800× *g* for 15 min at 4 °C. The supernatant was discarded, and the pellet was re-suspended in 0.1 M PBS (pH 7.2) [20].

### 2.5. Determination of Protein Entrapment Efficiency of Nanoparticles

The entrapment efficiency (E%) of the complex and total protein entrapped inside the per milligram of calcium phosphate nanoparticles was determined by releasing the bound protein in the calcium phosphate protein complex [22]. The CaP-Omp-Nps complex dissolved in 0.1 M PBS (pH 7.2) was centrifuged at 800× *g* for 20 min at 4 °C. The supernatant was removed, and the pellet was weighed and resuspended in 100 µL of 100 mM EDTA, then incubated at room temperature for 1 h. The suspension was centrifuged at 800× *g* for 20 min, and the supernatant was transferred to a fresh tube. The protein concentration of the released protein in the supernatant was analyzed by a spectrophotometric assay at 280 nm and by 2X dye on SDS-PAGE. The percentage efficiency of entrapment (E%) was then calculated from the amount of protein added initially in the preparation (Protein O) using the equation

E% = Protein R × 100/Protein O

where Protein R is the amount of protein released.

### 2.6. Determination of the Total Protein Loading Capacity of Nanoparticles

The total protein loaded onto the calcium phosphate nanoparticles is estimated by releasing the loaded protein after the cellobiose-mediated outer coating of nanoparticles (giving the amount of protein packed inside the nanoparticles plus protein coated outside the nanoparticles). The CaP-Omp-Nps complex dissolved in PBS was centrifuged at 800× *g* for 20 min, and the resulting pellet was weighed. The protein was released from the CaP-Omp-Nps using the method described above. The released protein was analyzed by measuring the optical density of the protein solution at the wavelength of 280 nm using the formula

L = Pr × 100/Cw

where Pr = amount of protein released (mg), Cw = total weight of the CAP-rOmp87 complex (mg).

The amount of protein loaded outside per mg of nanoparticles was obtained by subtracting the protein entrapped inside per mg of nanoparticles (calculated by deducing the entrapment efficiency) from the total protein loaded per mg of nanoparticles [22].

### 2.7. Toxicity Analysis of Calcium Phosphate Adjuvanted Omps Nano-Vaccine Formulation

The toxicity of CaP-Omp-Nps was tested in Wistar rats of both sexes (6–8 weeks old). Rats were obtained from the Indian Veterinary Research Institute (IVRI), Izatnagar, India. They were housed in the institutional small-animal experimentation facility for 1 week for acclimatization and were used for toxicity testing after approval from the Institute Animal Ethics Committee, vide approval number IAEC/CVASc/VPT/373. During acclimatization, rats were screened for *Salmonella* infection by PCR and monitored for any clinical signs and symptoms. The rats were divided into two groups, each consisting of six rats. Group I rats received a subcutaneous injection of 50 µg (50 µL) of CaP-Omp-Nps, while Group II (control group) received 50 µL of 0.01 M PBS (pH 7.4). The rats were observed for any signs of acute toxicity, such as nausea, vomiting, lack of appetite, sedation, or convulsions. Blood samples were collected at 72 h and on the 14th day post-treatment. Each sample was divided into two parts: one for hematological analysis, including hemoglobin (Hb), total leukocyte count (TLC), differential leukocyte count (DLC), and packed cell volume (PCV), to evaluate the nanoparticles’ hemotoxic effects. Serum was collected from the second portion of the blood sample to assess liver and kidney markers and evaluate potential hepatic or nephrotoxicity [23].

### 2.8. Testing of the Immune Potential of the Nano-Vaccine Formulation in Poultry Birds

The immune potential of the vaccine formulation was tested in poultry birds. Specific pathogen-free eggs of White Leghorn birds were procured from Venky’s India Ltd., Pune, India, and the eggs were hatched at the Instructional Poultry Farm. Chicks were reared under strict hygienic conditions in the animal experimental rooms of the Department of Veterinary Microbiology, College of Veterinary and Animal Sciences, Pantnagar, as approved by the Institute Animal Ethics Committee, vide approval number IAEC/CVASc/VPT/260 and IAEC/CVASc/VPT/373. The birds were provided with food and water ad libitum. The birds were tested for the presence of *Salmonella* in their feed samples using PCR. The birds (4 weeks of age) were divided into three groups, each comprising 15 birds: the control, calcium phosphate nanoparticle, and Montanide groups. Birds of the control group were injected with 100 µL of PBS subcutaneously (S/C), calcium phosphate nanoparticle group birds were vaccinated (S/C) with 100 µg of calcium phosphate-Omps complex in 100 µL PBS, and birds of the Montanide group were vaccinated (S/C) with 100 µg of Omps in 50 µL PBS with the same volume of Montanide. Each group received a booster dose at the same dose after 21 days following the first infection. Birds were bled on the 15th, 30th, and 45th day after the booster. Serum was collected and used to assess antibodies by enzyme-linked immunosorbent assay (ELISA).

### 2.9. Assessment of Humoral Immune Response by Enzyme-Linked Immunosorbent Assay (ELISA)

ELISA was used to assess antibodies, following the method described by Jha et al. (2015) with a few modifications [24]. In brief, ELISA plate wells were coated with approximately 1 µg Omps (100 µL) coating buffer, and plates were incubated at 37 °C for 2 h. The plates were washed twice with phosphate-buffered saline containing 0.05% Tween-20 (PBST) and blocked with 2% bovine serum albumin (BSA) in phosphate-buffered saline, pH 7.4, at 4 °C overnight. The plates were washed twice with PBST. The serum from each sample was diluted 1:100. Each row was assigned to a single diluted serum (1:100) sample, and two-fold dilutions were prepared in subsequent wells of the row. Plates were incubated at 37 °C for two hours and then washed with PBST. Then, 100 μL of diluted (1:2500 in Phosphate buffer) secondary antibodies (rabbit anti-chicken IgG, Sigma) were added to each well. The plates were incubated at 37 °C for 1 h and then washed three times with PBST. Then, 100 μL of substrate buffer (0.1 M Citric acid, 0.2 M dibasic sodium phosphate, 40 mg orthophenylene diamine (Sigma), and 40 µL of hydrogen peroxide, with a total volume of 10 mL of water) was added to each well, followed by incubation for one hour. The optical density was measured at 492 nm, and the titre was calculated for the Montanide and calcium phosphate groups relative to the control wells.

### 2.10. Delayed-Type Hypersensitivity (DTH) Assay

For assessing the cell-mediated immune response, the DTH assay was considered the test of choice. The DTH assay in birds was performed using the footpad swelling method with a few modifications [15]. Birds of all groups received 100 µg (50 µL) Omps in the left foot, while the right foot was injected with 0.1 M phosphate-buffered saline to equal volume (50 µL). The swelling in the footpad was observed and measured by Vernier calipers at different time intervals. The mean values of swelling in the right foot were subtracted from the values of swelling in the left foot, and the mean value of swelling in each group. The DTH graph was prepared by plotting the mean swelling values at different time intervals.

### 2.11. Assessment of Protective Immunity by Challenge Studies

Birds from all groups were challenged orally with 10^7^ CFU on the 45th day after the last booster. The birds of all three groups were monitored for mortality in the next two weeks. Salmonella shedding was assessed by PCR using cloacal samples from each group at 1-, 3-, and 5-day intervals post-challenge. After completion of the experiment, all the birds were euthanized ethically, and the carcasses were disposed of by the deep burial method.

### 2.12. Statistical Analysis

The statistical analyses were performed at the significance threshold of *p* < 0.05. and *p* < 0.01 by Student’s *t*-test using Graph Pad Prism v.8.0 for Windows (Graph Pad Software, San Diego, CA, USA). A two-factor ANOVA was used to calculate *p*-values comparing the control and vaccinated groups to assess toxicity and protective immunity.

## 3. Results

### 3.1. Isolation and Protein Profiling of Omps of S. Typhimurium

**Figure 1:**
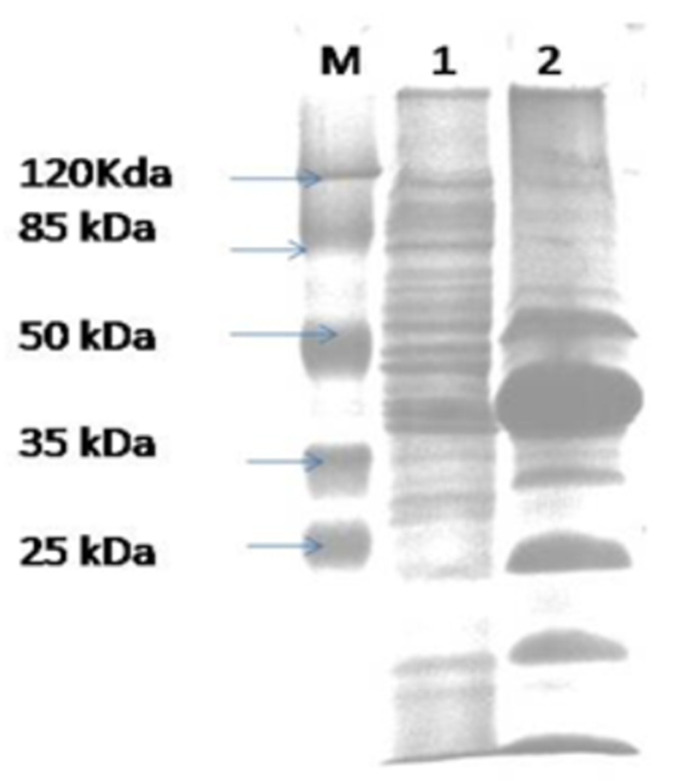
Protein profiling of outer membrane proteins of *Salmonella* Typhimurium (MTCC 3231) Lane M: Protein molecular weight marker, Lane 1: Whole cell protein profile of *S.* Typhimurium MTCC 3231, Lane 2: Protein profile of Omps of *S.* Typhimurium MTCC 3231.

Outer membrane proteins (Omps) of *S*. Typhimurium MTCC 3231 were isolated and compared with the whole cell proteins. SDS-PAGE (lane 1 of Figure 1) shows several bands in the entire protein extract. The molecular weight of Omps ranged from low to high. Several bands were observed in SDS-PAGE, among which five were prominent, corresponding to 20, 25, 32, 38, and 50 KDa (Figure 1). After quantification of Omps by Lowry’s method, the yield of Omps was found to be approximately 30 mg/L. After purifying and quantifying Omps, 1 mg of Omps was used for nanoparticle synthesis. The nanoparticles were characterized by their size, shape, stability, protein entrapment efficiency, and protein loading capacity.

### 3.2. Determination of the Shape and Size of Nanoparticles

**Figure 2:**
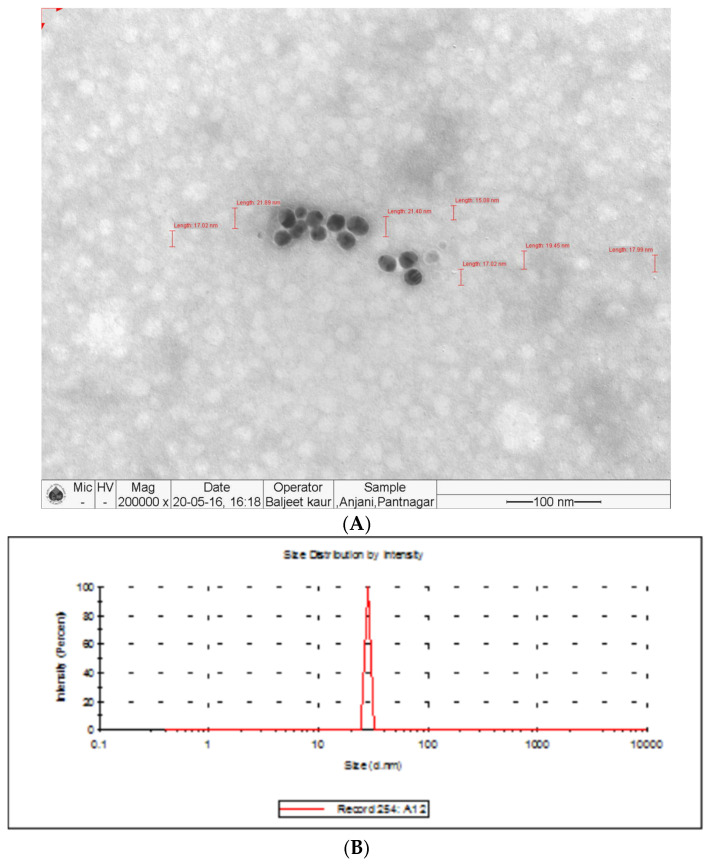
(**A**) TEM micrograph of calcium phosphate nanoparticles. The average particle size was observed to be in the range of 20–30 nm at 200,000× magnification. (**B**) DLS spectrum of calcium phosphate adjuvanted outer membrane nanoparticles. The peak corresponded to particle size of 45 nm.

Transmission electron microscopy was used for the determination of the shape of CaP-Omp-Nps. In transmission electron microscopy, nanoparticles exhibited an oval shape. The size of the particles ranged from 20 to 30 nm. The dynamic light scattering analysis showed a single sharp peak corresponding to an average size of 45 mm (Figure 2A,B). Electron microscopy and dynamic light scattering analysis confirmed that the size of the nanoparticles was less than 50 nm.

### 3.3. Characterization of CaP-Omp-Nps for Their Stability

**Figure 3:**
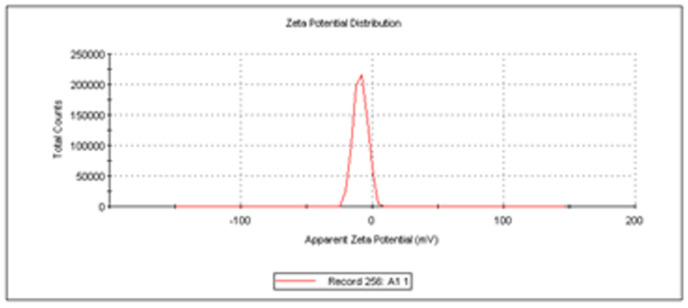
Zeta potential analysis of Calcium phosphate-Omps nanoparticles. A single sharp peak indicated stable nanoparticles with a zeta potential of −9.07 mV.

The stability of CaP-Omp-Nps was determined by estimating the zeta potential of particles. The zeta potential of the particles (Figure 3) was −9.07 mV, which indicated a satisfactory stability of the anionic nanoparticle complex.

### 3.4. Determination of Protein Entrapment and Protein Loading Capacity of Nanoparticles

The protein entrapment capacity was deduced by releasing the protein with EDTA treatment and was found to be 42.50%. After coating with cellobiose, the total loading capacity was 50.30%. The protein entrapped inside the nanoparticles was 40 µg/mg, and the protein coated outside the complex was 261 µg/mg of calcium phosphate-Omp Complex.

### 3.5. Assessment of the Toxicity of Nanoparticles

Acute toxicity of CaP-Omp-Nps was assessed by subcutaneous injection in Wistar rats. After the injection, there was no sign of toxicity such as vomiting, nausea, restlessness, lack of appetite, sedation, or convulsions. There was no granuloma, abscess, or ulceration at the site of injection. There was no mortality of rats any group (Test or control) till the termination of the experiment.

### 3.6. Effects of Nanoparticles on Liver- and Kidney-Specific Markers

In biochemical analysis of liver-specific markers, Aspartate transaminase (AST) and Alanine transaminase (ALT) were measured in the control and test group (CaP-Omp-Nps) at 72 h and 14 days after the injection. At 72 h and 14 days, aspartate aminotransferase (AST) levels in the control and treatment groups did not differ significantly. For alanine aminotransferase (ALT), there was also no significant difference in mean ALT activity between the control and treatment groups at 72 h and 14 days. The ALT values in both groups were well within the normal range. At 72 h and at 14-day intervals, the values of total bilirubin were found to be well within the normal range. The findings did not reveal any hepatotoxicity. In the kidney function test, the values for blood urea (BUN) and creatinine were well within normal limits at 72 h and 14 days. At 14 days, the BUN values in both groups decreased slightly, but the reduction was not significant and remained well within the normal range. The total protein values in both groups were well within the normal range during the toxicity studies. Among all liver and kidney function markers, there was no significant difference between the test and control groups (*p* value > 0.05) (Table 1a,b), suggesting the safety of nanoparticles in toxicity testing at 72 h and 14 days in Wistar rats.

### 3.7. Effects of CaP-Omp-Nps on Hematological Parameters

The effect of nanoparticles on hematological parameters was evaluated, and the findings indicated no significant differences among the parameters between the treated and control groups. No significant differences were observed in Hemoglobin, total leucocyte count, or differential leucocyte count between the two groups. Erythrocytes sedimentation rate (ESR) and packed cell volume (PCV) were well within the normal range in rats of both groups at 72 h and 14 days intervals (Table 2a,b). Based on hematological findings, the calcium phosphate-Omps nanoparticle complex was deemed safe in toxicological evaluation study in rats.

### 3.8. Testing of the Immune Potential of Vaccine Formation

After coating with cellobiose, CaP-Omp-Nps were tested for their immunogenicity in a poultry model. The vaccine was tested for humoral immune response (ELISA) and cell-mediated immune response by challenge studies.

### 3.9. Delayed-Type Hypersensitivity Assay

**Figure 4:**
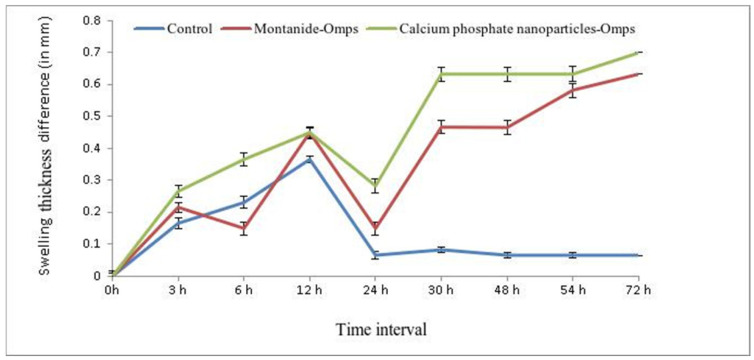
Footpad swelling pattern observed by DTH assay in the vaccinated groups (CaPNps-Omps nanoparticles and montanide-Omps) and control group of poultry birds at different time intervals (n = 18, six birds in each group). Foot pad swelling was not significant in the mice of control group but in both of the vaccinated groups foot pad swelling sustained till 72 h. Both of the vaccinated groups exhibited a significantly delayed type of hypersensitivity reaction.

Birds from all three groups (Control, Montanide-Omps, and CaP-Omp-Nps) were used to assess delayed-type hypersensitivity. Both vaccinated groups (Montanide-Omps and CaP-Omp-Nps) produced delayed-type hypersensitivity, but the response in the CaP-Omp-Nps group was stronger than in the Montanide-Omps group. Swelling was induced in all three groups within three hours and increased at 6 and 9 h. However, the magnitude of swelling was highest in the CaP-Omp-Nps group. After 24 h, the swelling in the control group had returned to nearly normal. In contrast, in the vaccinated groups, it remained elevated, increased further after 24 h, and was sustained even after 72 h (Figure 4). These findings confirmed the efficacy of calcium phosphate-Omps nanoparticles in eliciting a cell-mediated immune response.

### 3.10. Assessment of Humoral Immune Response by ELISA

**Figure 5:**
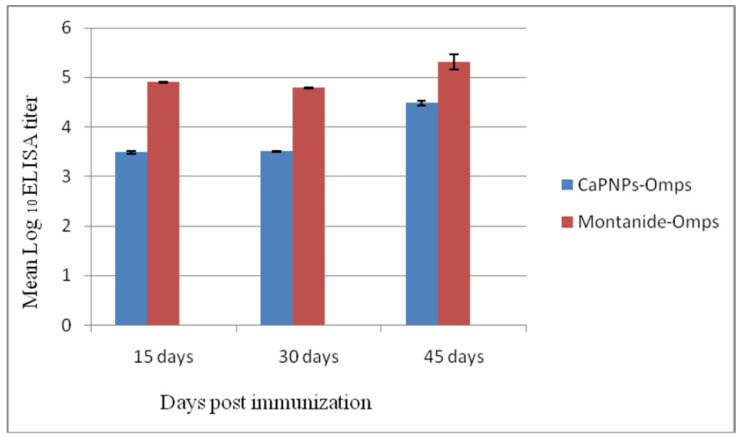
Mean titer (Log_10_ value) observed in different vaccinated and control groups of poultry birds (n = 45, 15 birds in each group) by ELISA. The mean titers with standard errors for the CaP-Omp-Nps and montanide-Omps groups were 3.48 + 0.0245 and 4.9 + 0.0142 on the 15th day, 3.5 + 0.0118 and 4.79 + 0.009 on the 30th day, and 4.48 + 0.0427 and 5.31 + 0.154 on the 45th day post-vaccination, respectively. ELISA titer of both the vaccinated groups was improved with the time interval indicating a strong and stable humoral immune response.

To assess the humoral immune response, IgG levels were measured in all three groups on days 15, 30, and 45 after the last booster [24]. In the Montanide group, a strong titer developed within 15 days and was sustained through day 45, with a slight reduction relative to the 15th-day titer. In the CaP-Omp-Nps group, the titer was not as strong on day 15, but it improved on days 30 and 45. A possible explanation is that Montanide is a sound oil-based adjuvant system that efficiently induces a humoral response immediately after the booster, whereas CaP-Omp-Nps releases antigen in two phases. Initially, Omps coated on the outside of the cellobiose are released, and in the second phase, Omps coated inside the nanoparticles are released and sustained. This is why the titer of Calcium phosphate-Omps adjuvanted nanoparticles was initially low, but over time it improved and became comparable to the Montanide group (Figure 5).

### 3.11. Assessment of Protective Immune Response

The protective immune response was evaluated by challenging all three groups with a strain of *S.* Typhimurium. Although no mortality occurred in any group, birds in the control group developed symptoms such as diarrhea. Bacterial shedding was assessed through *Salmonella*-specific PCR on cloacal swabs. In the control group, all birds shed *Salmonella* on days 1, 3, and 5 post-challenge. In the montanide group, 40% of birds shed bacteria on day 1 and 50% on days 3 and 5. In the CaP-Omp-Nps group, 30% shed *Salmonella* on day 1, increasing to 40% on days 3 and 5. Both vaccinated groups showed reduced *Salmonella* shedding (Table 3).

## 4. Discussion

The poultry sector has experienced rapid growth globally in recent years. India ranks second in egg production and fifth in poultry meat production, but it has not fully capitalized on this growth due to issues such as *Salmonella* contamination in poultry eggs. Additionally, the risk of *Salmonella* transmission from poultry products to humans remains a concern, as contaminated products can cause illness [25]. Nanotechnology offers new options for adjuvants that generate balanced Th1 and Th2 immune responses. Calcium phosphate nanoparticles have been used as adjuvants in vaccines against human and veterinary pathogens; they are biodegradable, cost-effective, and scalable for large-scale vaccine production [26]. Thus, calcium phosphate nanoparticles have been chosen as an adjuvant. In our study, the size of CaP-Omp-Nps fell within the recommended range for nanoparticles used in drug and vaccine delivery, consistent with previous research [16,27,28,29]. Nanoparticle stability, crucial for vaccine efficacy, was indicated by favorable zeta potential values, similar to earlier findings [16,30]. Zeta potential of CaP-Omp-Nps was found to be −9.07 mV, which reflected a satisfactory stability of nano-particles in the colloidal system. Although calcium phosphate nanoparticles tend to clump via non-ionic interactions in colloidal systems, once these formulations are injected into the host body, they are taken up by antigen-presenting cells. Antigen-presenting cells further process the entrapped antigens via endogenous antigen processing and present the processed antigens on their surface through MHC molecules to activate cell-mediated and humoral immune responses. Particle-size antigen-entrapping efficacies are critical factors in the induction of immune responses by antigen-presenting cells. In our study, the nano-vaccine formulation met both criteria (Particle size and antigen entrapment efficacy). EDX analysis confirmed the purity, crystalline structure, and successful entrapment of Omp proteins in calcium phosphate-adjuvanted nanoparticles. These particles demonstrated high protein entrapment and loading efficiencies, essential for effective antigen delivery, comparable to those reported in previous studies on calcium phosphate as a protein antigen adjuvant [15,16,31]. Calcium phosphate nanoparticles have been used to deliver various antigens, including nucleic acids and proteins, although the exact molecular-level mechanism of immune induction remains poorly understood. It is believed that they trigger immune responses via three mechanisms: the depot effect, NLRP-3 inflammasome activation, and enhanced antigen uptake and efficient presentation, resulting in improved Th1 and Th2 responses necessary for humoral and cell-mediated immunity [32]. In our study, vaccinated birds showed stronger cell-mediated immune responses with CaP-Omp-Nps compared to the Montanide-Omp vaccine group, similar to observations in mammalian models [16]. In the humoral response, the Montanide-Omps group elicited a faster, stronger response, while the CaP-Omp-Nps group showed a gradual increase. This is likely because the initial antibody response in the CaP-Omp-Nps group was generated by nanoparticle-surface antigens, with subsequent release of Omps that amplified and stabilized antibody production, potentially offering long-term protection against *Salmonella* [33]. Birds were challenged on day 45 post-last booster, and a reduction in *Salmonella* shedding served as a measure of protection. Both vaccinated groups, Montanide-Omps and CaP-Omp-Nps, significantly reduced shedding compared to controls, with the calcium phosphate-Omps group showing slightly better protection, aligning with earlier reports [16]. Although Montanide is an efficient oil-based adjuvant, it can cause tissue reactions like swelling and pain at the injection site [34]. Our study found no such adverse tissue reactions with CaP-Omp-Nps, corroborating previous findings [35]. The immune responses induced by the nano-formulation were comparable to Montanide, making CaP-Omp-Nps a potential alternative to oil-based adjuvants. Safety assessments showed no significant effects on hematology, liver, or kidney functions, with nanoparticle-injected groups resembling controls—consistent with prior studies [16,35,36]. These results suggest that nano-vaccine formulations are safe and may be suitable for delivering *Salmonella* antigens.

## 5. Conclusions

Based on our findings, the CaP-Omp-Nps vaccine formulation is deemed safe as no significant toxicity symptoms could be observed in the study in rat model. The values of kidney- and liver-specific markers were found well within the normal range. It stimulated both humoral and cell-mediated immune responses and reduced *Salmonella* shedding. This suggests it may be proven an effective candidate for the development of a subunit vaccine against poultry salmonellosis. Our results lay the groundwork for testing the vaccine’s effectiveness on a larger scale and exploring the possibility of converting the injectable form into an easily administered oral version by surface-modifying the nanoparticles. Ultimately, this could lead to a vaccine suitable for large-scale use, benefiting the poultry industry.

## Figures and Tables

**Table 1:** (**a**) Comparison of liver- and kidney-specific markers values in blood serum collected from rats of control and Cap-Omp-Nps-treated group at 72 h intervals. (**b**) Comparison of liver- and kidney-specific markers values in blood serum collected from rats of control and Cap-Omp-Nps-treated group at 14-day intervals.

S.No.	Biochemical Parameters	Control Group (N = 6) (Mean ± SE) (SD)	Cap-Omp-Nps Treated Group *p* Value (N = 6) (Mean ± SE) (SD)
(**a**)			
1.	AST(U/L)	24.250 ± 0.70 (1.72)	24.51 ± 0.78 (1.91) (0.8051)
2.	ALT(U/L)	14.60 ± 1.26 (3.10)	22.6 ± 0.66 (1.63) (0.1932)
3.	BUN (mg/dL)	32.66 ± 2.34 (5.70)	37.16 ± 1.37 (3.37) (0.1292)
4.	Total Protein (g/dL)	8.53 ± 1.36 (3.53)	6.59 ± 0.87 (2.14) (0.2377)
5.	Creatinine(mg/dL)	0.68 ± 0.134 (0.32)	0.78 ± 0.080 (0.107) (0.5649)
6.	Total Bilirubin(mg/dL)	0.73 ± 0.040 (0.098)	0.94 ± 0.00774 (0.18) (0.468)
(**b**)			
1.	AST (U/L)	24.2 + 1.54 (3.79)	24.11 ± 1.17 (2.87) (0.9599)
2.	ALT(U/L)	13.5 + 1.80 (4.41)	11.7 ± 0.97 (2.39) (0.4008)
3.	BUN (mg/dL)	25.4 ± 0.16 (0.04)	24.7 ± 1.49 (3.65) (0.6823)
4.	Total Protein (gm/dL)	7.0 ± 0.38 (0.95)	6.4 ± 0.20 (0.51) (0.2410)
5.	Creatinine (mg/dL)	1.65 ± 0.174 (0.43)	1.58 ± 0.160 (0.39) (0.7841)
6.	Total Bilirubin (mg/dL)	0.79 ± 0.089 (0.22)	0.87 ± 0.016 (0.040) (0.4178)

AST: Aspartate Transaminase; ALT: Alanine Transaminase; BUN: Blood Urea Nitrogen, SD: standard deviation SE: Standard error. (**a**) Serum biochemical indices in rats after 3 days of injection in control and Cap-Omp-Nps vaccinated groups. The results are expressed as mean ± SE (Standard error) values for each parameter analyzed. The findings indicated no significant changes (*p* < 0.05) in the CaP-Omp-Nps complex-treated group of rats compared with the control group of rats. (**b**) Serum biochemical indices in rats after 14 days of injection in control and Cap-Omp-Nps vaccinated groups. The results are expressed as mean ± SE (Standard error) values for each parameter analyzed. The findings indicated no significant differences (*p* < 0.05) between the CaP-Omp-Nps-treated group and the control group of rats.

**Table 2:** (**a**) Comparison of Hematological parameters in blood samples collected from rats of control and Cap-Omp-Nps-treated groups of rats at 72 h intervals. (**b**) Comparison of Hematological parameters in blood samples collected from rats of control and Cap-Omp-Nps-treated groups of rats at 14-day intervals.

(a)			
S.No.	Parameters	Control Group (N = 6) (Mean ± SE) (SD)	Cap-Omp-Nps Treated Group (N = 6) *p* Value (Mean ± SE) (SD)
1.	Hemoglobin (g/dL)	19.9 ± 0.571 (1.40)	18.9 ± 1.11 (2.73) (0.4585)
2.	Total leucocyte count (TLC) (thousand cells/µL of blood	4608 ± 99.51 (243.75)	4558 ± 225.24 (0.551.75) (0.84332)
3.	Differential leucocyte count (DLC) (%)	35.1 ± 2.41 (N) (5.91) 63.8 ± 2.227 (L) 1.16 ± 0.3072(M)	30.6 ± 1.92 (N) (1.92) (0.1758) 70 ± 1.949 (L) 0.83 ± 0.307 (M)
4.	Erythrocyte sedimentation rate (ESR) (mm/hr)	0.15 ± 0.0220 (0.054)	0.15 ± 0.022 (0.055) (1.000)
5.	Packed cell volume (PCV) (%)	37.1 ± 1.376 (3.371)	39.16 ± 1.939 (4.750) (0.3879)
(**b**)			
**S.No.**	**Parameters**	**Control (SD)**	**CaP-Omp-Np** **Treated** **Group (SD)** ***p*** **Value**
1.	Hemoglobin (g/dL)	16.6 ± 0.467 (1.145)	16.51 ± 0.275 (0.676) (0.8811)
2.	Total leucocyte count (TLC) (thousand cells/µL of blood	5175 ± 88.99 (218)	5450 ± 195.14 (478) (0.2230)
3.	Differential Leucocyte count (DLC) (%)	37.50 ± 0.8940 (N) 61.16 ± 1.973 (L) 2.50 ± 0.223 (M)	37.83 ± 1.720 (N) (0.8940) 57.33 ± 2.389 (L) 1.5 ± 0.223 (M)
4.	Erythrocyte sedimentation rate(ESR) (mm/hr)	0.28 ± 0.030 (0.0752)	0.30 ± 0.036 (0.089) (0.7342)
5.	Packed cell volume (PCV) (%)	47.33 ± 0.662 (1.632)	47.1 ± 1.376 (3.371) (0.9154)

N: Neutrophils, L: Lymphocytes, M: Monocytes, SD: standard deviation SE: Standard error. (**a**) Hematological parameters are expressed as mean ± SE values for each parameter analyzed. The findings indicated no significant changes in various hematological parameters (*p* < 0.05) in the blood samples of CaP-Omp-Nps-treated rats compared with the control group at 72 h intervals. (**b**) Hematological parameters are expressed as mean ± SE values for each parameter analyzed. The findings indicated no significant changes (*p* < 0.05) in blood samples from CaP-Omp-Nps-treated groups of rats compared to the control group at 14-day intervals.

**Table 3:** Shedding of *Salmonella* in fecal samples of birds in different groups at different time intervals after challenge with 10^7^ CFU of a virulent strain of *Salmonella* Typhimurium tested by *Salmonella* specific PCR.

Groups	Number of Birds Found Positive at Day One (Birds Shedding/Total Number of Birds)	Number of Birds Found Positive at Day Three (Birds Shedding/Total Number of Birds)	Number of Birds Found Positive at Day Five (Birds Shedding/Total Number of Birds)
Control Group	10/10	10/10	10/10
Montanide Group	4/10	5/10	5/10
CaP-Omp-Nps group	3/10	4/10	4/10

A significant difference was observed between the control and vaccinated groups (Montanide Group and Cap-Omp-Nps group) in a two-factor ANOVA (*p*-value = 0.000403).

## Data Availability

No data from the study was submitted to thedata bank of NCBI.
